# Counting distance: Effects of egocentric distance on numerical perception

**DOI:** 10.1371/journal.pone.0174772

**Published:** 2017-04-14

**Authors:** Nurit Gronau, Anna Izoutcheev, Tsafnat Nave, Avishai Henik

**Affiliations:** 1 Department of Psychology and Cognitive Science Studies, The Open University, Raanana, Israel; 2 Department of Psychology and Zlotowski Center for Neuroscience, Ben-Gurion University of the Negev, Beer-Sheva, Israel; Centre de neuroscience cognitive, FRANCE

## Abstract

Numerical value is long known to be associated with a variety of magnitude representations, such as size, time and space. The present study focused on the interactive relations of numerical magnitude with a spatial factor which is dominant in everyday vision and is often overlooked, namely, egocentric distance, or depth. We hypothesized that digits denoting large magnitudes are associated with large perceived distances, and vice versa. While the relations of numerical value and size have been long documented, effects of egocentric distance on numeral perception have been scarcely investigated, presumably due to the difficulty to disentangle size and depth factors within three-dimensional visual displays. The current study aimed to assess the potential linkage between egocentric distance and number magnitude, while neutralizing any perceived and/or physical size parameters of target digits. In Experiment 1, participants conducted a numeral size-classification task (‘bigger or smaller than 5’), to which they responded with a near-to-body or a far-from-body key. Results revealed shorter responses for small than for large numbers when responded with a key positioned close to the body, and for large than small numbers when responded with a key positioned far from the body (regardless of hand-key mapping). Experiment 2 used verbal stimuli denoting near/remote concepts as irrelevant primes to target digits, further demonstrating a priming effect of conceived distance on numerical value processing. Collectively, our results suggest that distance magnitudes are associatively linked to numerical magnitudes and may affect digit processing independently of the effects of visual size.

## Introduction

The use of digits—symbolic entities denoting quantities or magnitudes–is a unique ability of human cognition. Various magnitude dimensions were found to be associated with digit processing, such as size, time and space [1,2]. Within the spatial domain, numbers are typically thought to be represented on a left-to-right “mental line”, such that small numbers are represented on the left of space, while large numbers are represented on its right [3–5]. Supportive evidence for this spatial representation comes mainly from studies demonstrating the well known *spatial-numerical association of response codes* (SNARC) effect, according to which large numbers are responded to faster than small numbers with right-sided responses, whereas an inversed pattern is observed when the response is executed on the left [4]. Furthermore, several studies have shown that numbers may not only be represented on a left-to-right horizontal plane, but also on a bottom-to-top vertical one [6–9].

Most studies investigating numeral perception, and specifically, the relations between number value and spatial representation, have used two-dimensional displays in which participants responded to a digit presented on a fixed horizontal or vertical plane. Here we propose an associative relation between numeral value and an additional dimension in space, namely, egocentric distance, or depth. Specifically, we propose that, in situations in which depth is a meaningful factor (e.g., a three-dimensional world), numbers may be represented on a close-to-far mental plane, where small numbers are associated with close locations, and large numbers are represented farther away from oneself and are associated with large egocentric distances.

A major difficulty in examining the effects of egocentric distance on numeral perception is the fact that the former is tightly linked to stimulus size: in a three-dimensional world, the physical size of stimuli and their perceived depth are negatively correlated, such that the greater the distance of an object from oneself, the smaller is its retinal size. Indeed, in the context of numeral cognition, one of the most studied factors affecting numeral perception has been the size of stimuli. In the well-known *size congruity effect* (SiCE), for instance, strong interactive relationships are observed between the physical size of a digit and its numerical value [1,10–14]. Specifically, when participants are required to decide which of two digits is numerically larger, responses in the number comparison task are affected by the physical size of the stimuli: responses are faster when the two digits denote numerical values which are congruent with their relative physical sizes (e.g., 8 2) than when the digits denote numerical values which are incongruent with their relative physical sizes (e.g., the digits 8 2). Effects of size on numerical processing have also been found in situations in which the perceived size, rather than the physical (i.e., retinal) size of stimuli was a key factor in the experimental manipulation. Using a “Ponzo Illusion” in which stimuli of equal retinal size are typically perceived as larger when presented at a distant location, Goldfarb and Tzelgov [15] showed a size congruity effect similar to the one obtained with stimuli of physically different sizes. Their findings suggested that perceived size, not necessarily physical size, may play a critical role in size-number interaction effects. Relatedly, when using paradigms in which images of real world objects were presented with equal retinal size, several studies have shown effects of conceptual size (i.e., semantic knowledge of an object’s size in real life) on numerical perception. Thus, for instance, in a parity judgment task performed on digit targets, an irrelevant prime image of a conceptually small (snail) or a large (horse) animal (both images presented at the same retinal size) facilitated responses for a small or a large target digit, respectively [16,17]. Taken together, these findings underscore the tight linkage between size and numerical representations.

As mentioned above, an important factor that is often overlooked and is strongly correlated with size perception is the perceived depth of an object: perspective depth cues allow the cognitive system to estimate an object’s real world size, contributing to size constancy perception [18]. That is, although the object’s retinal size decreases as its egocentric distance increases we perceive the object’s size as constant. Importantly, when examining size-number associations, the perceived depth of a stimulus (e.g., digit) may affect numeral perception and may partly account for the interactive relations between size and numerical value dimensions. Thus for instance, a digit presented at the far end of a three-dimensional display (ponzo illusion) is perceived as bigger, as well as more distant, than a digit of equal retinal size presented at the near plane of the display. It is possible, thus, that both size and distance factors contribute to the congruency effects observed in a numeral comparison task [15]. Similarly, a large real world object (e.g., horse) serving as a prime in a parity judgment task [16] is likely perceived as viewed from a much larger distance than a small object (e.g., snail) of equal retinal size. When controlling for retinal size factors, thus, both perceptual as well as conceptual (real-world) size representations are confounded with the perceived distance of stimuli (i.e., both dimensions are big, or both are small). While ample evidence has established the strong linkage between physical size and number perception, in cases in which depth information serves as an important cue for object size computation, the perception of an object’s egocentric distance may partly account for the effects of its size on numeral perception. Perceived distance, thus, may serve as an important factor affecting numeral magnitude representation, above and beyond the effects of size, yet it is extremely difficult to assess its unique contribution due to the difficulty to disentangle the two factors.

The present study aimed to investigate the potential linkage between egocentric distance, or depth, and numerical value, via manual responses which are executed close or far from one’s body during the completion of a number-size classification task. Critically, in contrast to experiments using visual displays, in which depth is directly manipulated (e.g., ponzo illusion) or is implied by objects’ retinal size (e.g., stimuli of different real-world sizes), depth was not manipulated via visual perspective cues of any sort in the current study. Rather, it was manipulated via activation of response-codes associated with close and far distances, while neutralizing any perceived and/or physical size parameters of target digits, thus avoiding a potential size-distance confound. The relation between egocentric distance and numeral value was assessed, then, via a “SNARC-like” task in which embodied ‘action codes’ were potentially associated with small and big number values, not via the perceived distance of visually presented stimuli.

To anticipate, our findings provided strong support for our hypothesis, revealing shorter responses for small than for large numbers when responded to close to the body, and for large than small numbers when responded to far from the body. In a follow-up study, we further manipulated the relations of distance and numeral value via activation of conceptual (i.e., verbal) information associated with close and far distances. Taken together, our results provide evidence, for the first time to our best knowledge, for the associative linkage between distance and numerical perception.

## Experiment 1: Effects of near and far responses on numerical size processing

### Method

#### Participants

Thirty-two undergraduate students from The Open University of Israel participated in the study for course credit (24 females, mean age = 25.7 years, SD = 4.6). The experiment has been approved by the Israel's Open University Ethical Review Board committee. All participants signed a consent form indicating that participation was voluntary and that they could withdraw from the experiment at any time without penalty.

#### Apparatus, stimuli and design

Stimuli were presented on a 15-in. monitor with an 85-Hz refresh rate. In each trial a black Arabic digit appeared at the screen center and participants were instructed to respond to its numerical size (smaller/bigger than 5) as fast as possible while maintaining accuracy. Digits denoted small (1,2,3) or large (7,8,9) values, each spanning approximately 1.1°x 1.8°. Critically, in contrast to typical experimental settings, the keyboard which collected participants’ responses was positioned vertically to the participants, rather than horizontally to them. Namely, it was rotated 90°clockwise and placed on the experimental table such that its rightmost part was positioned close to the participant’s body (aligned with the table’s edge), while its leftmost part was positioned far from the participant. Two keys, one on the rightmost part (i.e., close end) and one on the leftmost part (far end) of the keyboard, were used for the number classification task (see detailed description below). We hypothesized that small numerical values would elicit faster RTs when responded with a close than with a far key, and vice versa for the big numerical values.

#### Procedure

Participants were seated approximately 50 cm from the screen. As mentioned above, in each trial they were asked to judge as fast as possible whether a digit was bigger or smaller than 5, by responding with their right and left hand. Two keys on an Apple keyboard (DirectIN Millisecond Accurate brand) were chosen for participants’ responses–the “Caps lock” key (at the leftmost side of the keyboard) and the “+” key (at the rightmost side of it, with a center-to-center distance of 42 cm between the two keys). Since the keyboard was rotated 90°clockwise (i.e., it was positioned vertically rather than horizontally), the latter key was close to one’s body while the former key required participants to stretch their arm far away from the body in order to respond. Note that in terms of the keyboard’s width, both response keys were aligned with each other and positioned approximately at the keyboard’s midpoint, thus preventing a potential response bias associated with the horizontal (i.e., left-right) plane while the keyboard was rotated vertically. The response keys were covered with colored stickers (green and yellow), and participants were instructed to respond to big and small numbers, according to the specified colors in each of the experimental parts (see below).

Each trial began with the appearance of a central fixation cross (0.3°) for 1000 ms. After a blank interval of 250 ms, a target digit appeared at the screen center, remaining until participants’ response to the digit classification task. An 1000 ms inter-trial interval (ITI) was inserted before the beginning of the next trial.

The experiment was divided into two parts: in one part, participants responded to “big” digits with their right hand and to “small” digits with their left hand, while in the second part of the experiment the response mapping was reversed (hands remained on the original keys, but responses flipped; the order of parts was counterbalanced across participants). In addition, to allow full counterbalancing of hands position and physical distance, for half of the participants the close key (i.e., “+”) was responded with the right hand while the far key (i.e., “Caps lock”) was responded with the left hand, and for the other half of the participants the hand-to-key mapping was reversed. This yielded a fully counterbalanced design, in which each participant had 50% of congruent trials (e.g., response to small numbers with a close key) and an additional 50% of incongruent trials (e.g., responses to small numbers with a far key).

Each experimental part consisted of two blocks. Within each block, the 6 digits were presented eight times, yielding a total of 48 trials per block, and an overall total of 192 trials in the experiment. The order of trials within each block were determined randomly. Prior to the beginning of each experimental part, participants completed two practice blocks, ensuring that that they were proficient with the response mapping of the corresponding part. As mentioned above, we hypothesized that small numerical values would elicit faster RTs when responded with a close than with a far key, and vice versa for the big numerical values.

### Results

**Fig 1:**
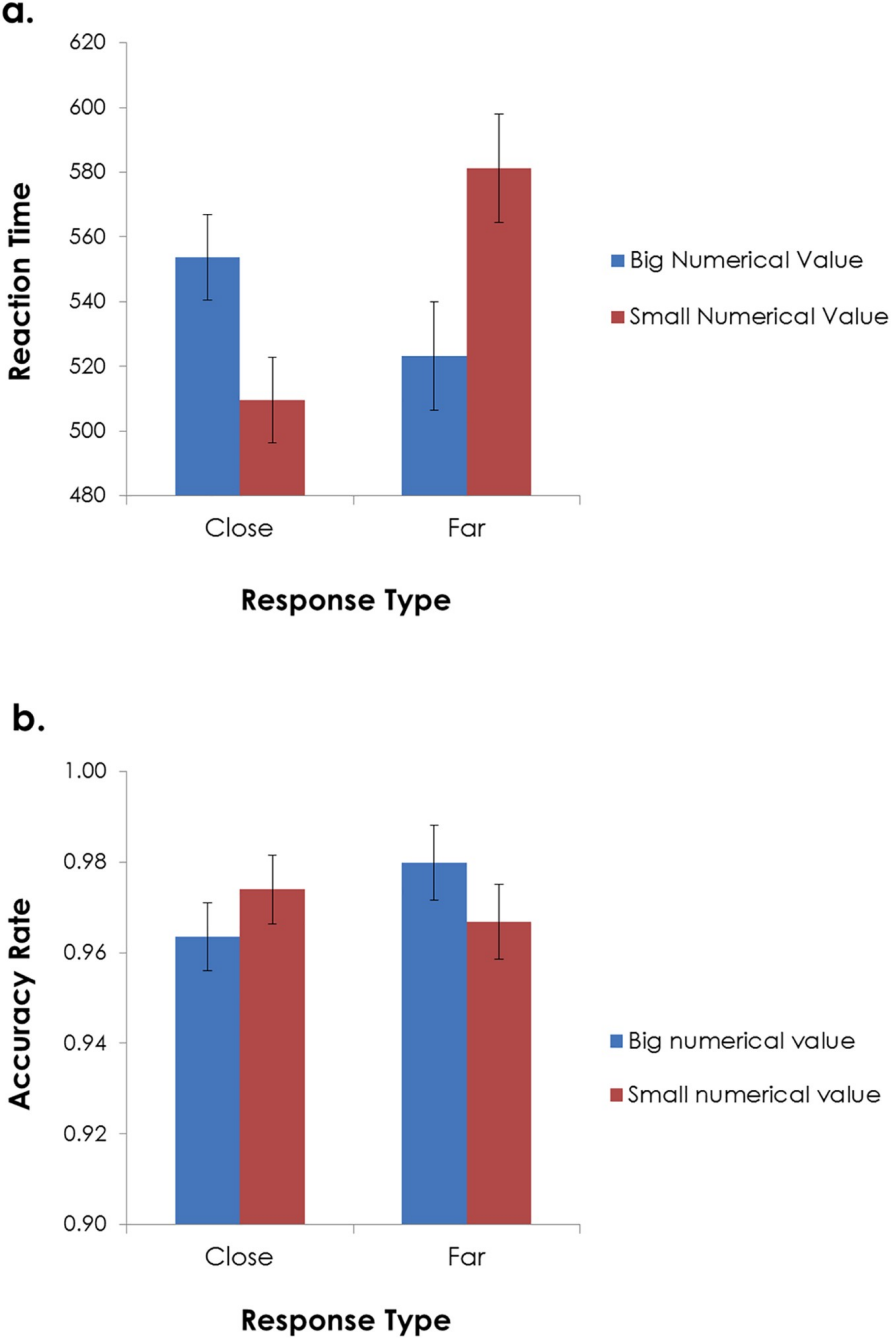
**Reaction times (a) and accuracy rates (b) in Experiment 1 as a function of response type (i.e., key distance) and numerical value.** Standard errors are computed for the difference score (between big and small numerical values) [19], within each of the response type conditions.

Accuracy rate was high in the number classification task and reached an overall level of 97% correct responses. Trials yielding extreme outlier responses (i.e., RTs that deviated from a participant’s mean RT by more than three standard deviations) were excluded from the RT analysis (2.1%). Fig 1 presents participants’ RTs as a function of response type and numerical value. A two-way analysis of variance (ANOVA) with response type (near key, far key) and target numerical value (small digits: 1–3, big digits: 7–9) as factors revealed a significant main effect of response type, F(1, 31) = 7.41, p < .02, η^2^_p_ = .19, indicating overall shorter response times for near than for far responses. There was no main effect of number size (F(1, 31) = 1.27, p > .1, η^2^_p_ = .04), however, a statistically significant interaction effect was obtained between the numerical size and the response mode (i.e., key distance) factors, F(1, 31) = 16.09, p < .0001, η^2^_p_ = .34. As hypothesized, the interaction stemmed from shorter responses for small than for big numbers when using the close key, F(1, 31) = 9.06, p < .005, η^2^_p_ = .23, and shorter latencies for big than for small numbers when using the far key, F(1, 31) = 10.56, p < .003, η^2^_p_ = .25).

Note that, as mentioned earlier, to allow full counterbalancing of hands and physical distance, for half of the participants the close key was responded with the right hand while the far key was responded with the left hand, and for the other half the response-mapping was reversed. To ensure that hand position could not account for the results, and/or did not have any influence on them, we conducted an additional three-way mixed Anova, in which hand position was inserted as a between-subject factor. This analysis replicated our previous findings (i.e., a statistically significant main effect of response type, F(1, 31) = 8.26, p < .01, η^2^_p_ = .22, along with a significant response type by numerical size interaction, F(1, 31) = 12.26, p < .001, η^2^_p_ = .29), yet there was no hint for a three-way interaction (F<1). All other main effects or interactions failed to reach significance level (p>0.1). Hand position, thus, had no effect on the numerical value by distance interaction effect observed.

In addition, we examined the potential hand-numerical size mapping effects on the reaction time data. Recall that in one part of the experiment participants responded to large numbers with their right hand and to small numbers with their left hand, while in the other part the hand-size mapping was reversed (with order of parts counterbalanced across participants). The division to the two experimental parts allowed us to examine the typical SNARC effect, in which right sided responses are normally associated with large numbers and left responses are associated with small numbers. This factor, however, could not be inserted as a within-subject factor in the three-way Anova described above, since it was not orthogonal to key distance (i.e., each participant had his/her right and left hands fixed on the two response keys across the two experimental parts, rather than being assigned to both hand-size mappings under each of the hand-key positions). We therefore analyzed the potential effects of hand-numerical size mappings by running a three-way Anova, in which numerical value and experimental part (i.e., hand-size mapping) were inserted as within subject factors, and the hand position was inserted as a between-subject factor. Note that the two hand positions (manipulated between participants) created two different states of affairs with respect to the relations between the Left-Right SNARC effect, and the Near-Far ‘SNARC-like’ effect discussed above. That is, when the right hand was positioned on the *far* key and the left hand was positioned on the close key, the two spatial-numerical mappings corresponded with each other: responding to big numbers with the right (far) hand was congruent for both Left-Right and Near-Far mappings, as was the response to small numbers with the left (close) hand. However, when placing the right hand on the *close* key and the left hand on the far key, the two types of mappings conflicted with each other. Namely, responding to big numbers with the right (close) hand was congruent from a Left-Right mapping perspective, but *incongruent* from a Near-Far mapping perspective (according to which close responses are associated with small numbers), and vice versa for small numbers. Therefore, we hypothesized that, to the extent that a Left-Right spatial-numeral mapping dominates the response pattern of results, a typical SNARC effect should be obtained in both hand-position groups (albeit, perhaps to a weaker degree in the latter group due to an opposing Near-Far mapping). If, in contrast, a Near-Far mapping is most dominant when using close and far response-keys, the two groups should elicit conflicting patterns of findings with respect to the Left-Right SNARC effect. In particular, the latter group (right = close) should reveal a pattern consistent with the Near-Far ‘SNARC-like’ phenomenon, and *not* with the typical Left-Right SNARC effect, resulting in a three-way interaction of the hand position factor with the numerical size and experimental-part (i.e., hand-size mapping) factors.

**Fig 2:**
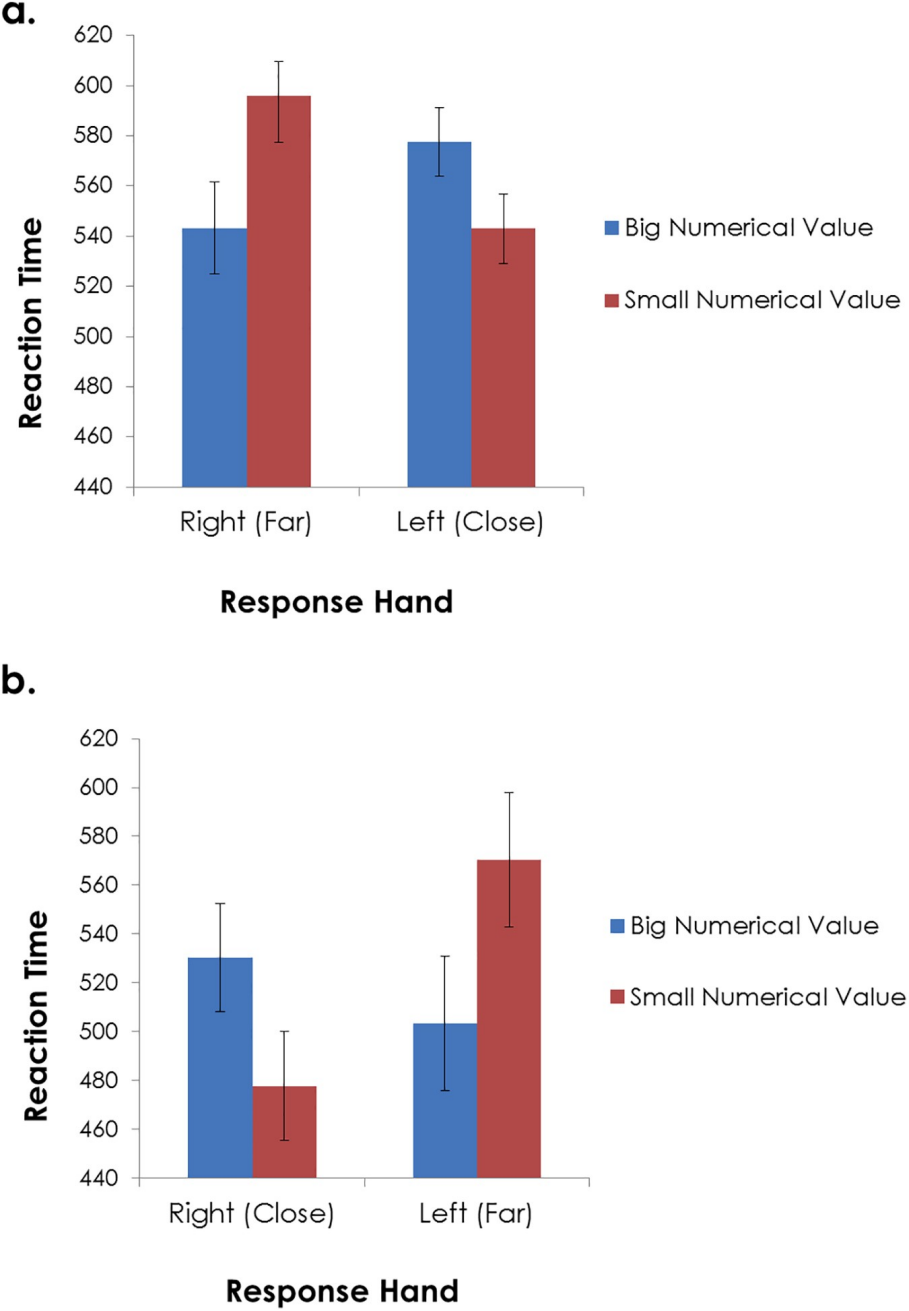
Reaction times in Experiment 1, as a function of response hand and numerical value, within each of the hand-position groups. In one group, the right hand was positioned on the far key and the left hand was positioned on the close key (a), while in the other group response keys were flipped (b). Standard errors are computed for the difference score (between big and small numerical values) [19], within each of the response hand conditions.

An Anova conducted on the RT data indeed revealed a statistically significant three-way interaction, F(1,30) = 7.75, p<0.01, η^2^_p_ = .21. To better understand the nature of this interaction we further conducted a two-way Anova within each of the hand-position groups. Fig 2 presents participants’ RTs as a function of hand (Right/Left) and numerical value (Big/Small), within each of the hand-position groups (N = 16). As can be clearly seen, when the right hand was positioned on the far key and the left hand was positioned on the close key (Fig 2A), a typical SNARC effect was obtained, F(1,15) = 14.47, p<0.005, η^2^_p_ = 0.49, stemming from shorter RTs to big than to small numbers responded with the right hand, and to small than big numbers responded with the left hand (t(15) = 2.86, 2.5, respectively; p<0.05 using Tukey post-hoc criterion). As explained above, this Left-Right spatial-numerical mapping was confounded with a Near-Far mapping. However, when the right hand was positioned on the close key and the left hand was positioned on the far key (Fig 2B), the results pattern flipped. Rather than obtaining the typical Left-Right SNARC effect, the statistically significant interaction supported a Near-Far spatial-numerical mapping, F(1,15) = 6.56, p<0.03, η^2^_p_ = 0.30. That is, responses were faster to big than to small numbers when responded with the left (far) hand, and were faster to small than to big numbers when responded with the right (close) hand (t(15) = 2.86, 2.5, respectively; p<0.05 using Tukey post-hoc criterion). Taken together, these findings strongly support a Near-Far spatial-numerical response mapping, which dominated a Left-Right mapping typically observed when participants are requested to respond to a central digit with left and right responses. It appears that when using a close-to-far response axis, rather than a left-to-right one, the latter axis becomes irrelevant to task requirements and is thus ineffective in its influence on number magnitude representation.

Finally, a two-way ANOVA including response type (distance) and numerical value was also conducted on the accuracy rates data, revealing a trend for an interaction in the hypothesized direction (see Fig 1B), however this interaction was only marginally significant, F(1, 31) = 3.18, p = .085, η^2^_p_ = .09. None of the main effects approached significance (all Fs < 1.2). Due to the high overall levels of accuracy and the non-significance of the results, no further analyses were conducted.

### Discussion

In a three-dimensional world, objects’ perceived size and distance are strongly interrelated within the visual modality. The present study aimed to assess whether distance is associated with number magnitude, independently of perceived size. To control for size factors, rather than manipulating distance via perceptual cues (such as visual perspective), we presented target digits at a fixed physical (and perceived) size on a computer screen, while manipulating distance via manual responses executed close or far from one’s body. In accord with our hypothesis, latency findings clearly demonstrated an associative linkage between numeral magnitude and egocentric distance, the latter experienced through one’s own bodily actions and sensations.

The tight relations between action and perception, and more generally, the importance of action ‘enactment’ and/or of embodied cognition, have long been recognized in the cognitive literature (for reviews, see e.g. [20–22]). Action-oriented representations have been shown to influence a wide diversity of tasks associated with perception, memory, knowledge, language, decision making, and attribution (see, e.g. [22]). Here, we used a “SNARC-like” task, in which action codes that represent the three-dimensional space surrounding one’s body were potentially activated, in association with numeral magnitude perception. Our findings revealed that manual responses executed close to the body were associatively linked to small numbers, while responses executed far from the body were linked to large numbers. These results imply that in a three-dimensional world, numbers are not only represented along a left-to-right or a bottom-to-top mental plane, but also along a near-to-far plane. Furthermore, the use of a near-far response axis clearly dominated number representation, as there was no hint for a left-right SNARC effect typically observed when using left and right responses.

Whereas response-mapping may serve as a powerful means for the assessment of associative space-number relations, one should nevertheless take into account a possible limitation of the current study. Although the target digits were presented at a fixed size on the computer screen, visual size parameters associated with participants’ hands position may have nonetheless affected responses to targets, as the space between one’s body and the hands differed between the two response modes. Namely, the visually perceived area between one’s own body and the far key was larger than the corresponding area for the close key, and this difference in area size may have contributed to the observed distance-number association. The sight of one’s hand stretched to respond far away, relative to the folded hand responding close to the body, may have additionally highlighted the differences between the two perceived area sizes.

While we cannot strictly rule out this account, we believe it is unlikely to explain our results, as participants focused their eyes on the visual display presented on the computer screen, rather than on their own hands and/or the area flanked by the hands. We nevertheless ran an additional study in which visual size parameters were excluded altogether, to allow a more “pure” investigation of distance-number associations. This study also allowed examining whether distance-number associations are necessarily grounded in one’s own bodily actions and sensations, or they may be additionally represented on a higher, more abstract, conceptual level.

Specifically, a priming paradigm was used, in which prime words denoting close and far concepts preceded target numbers. We hypothesized that the word “near” would mainly activate small numerical values, while the word “far” would activate large numerical values. Participants conducted a size-comparison task on the target digits, similar to the one conducted in Experiment 1, and their responses were analyzed as a function of prime type and target numeral size. Thus, rather than examining the relations between close/far actions and number size, distance was manipulated in Experiment 2 via verbal (i.e., conceptual) stimuli which were potentially linked to different number magnitudes.

## Experiment 2: Effects of “near” and “far” prime words on numerical size processing

### Method

#### Participants

Twenty-five undergraduate students from The Open University of Israel participated in the study for course credit (20 females, mean age = 28 years, SD = 5.8). The experiment has been approved by the Israel's Open University Ethical Review Board committee. All participants signed a consent form indicating that participation was voluntary and that they could withdraw from the experiment at any time without penalty.

#### Apparatus, stimuli and design

Prime words were displayed in Hebrew and represented either the word “near” (*karov*) or the word “far” (*rachok)*. The words, comprising 4 letters each, spanned approximately 3.3°by 1.8°. Target numbers consisted of black Arabic digits that denoted small (1,2,3) or large (7,8,9) values, each spanning approximately 1.1°x 1.8°. All other experimental parameters were identical to Experiment 1.

#### Procedure

Participants were seated approximately 50cm from the computer monitor. They were instructed to maintain fixation at the center of the screen throughout the experiment. Each trial began with the appearance of a central fixation cross (0.3°) for 1000 ms. After a blank interval of 250 ms, a prime word appeared for 250 ms. Following an additional blank interval of 500 ms, a target digit appeared until participants’ response. Participants were asked to ignore the word and to judge as fast as possible whether the digit was bigger or smaller than 5 while maintaining accuracy (using the “Z” and “M” keys, respectively). Both prime and target were presented at the screen center. An 1000 ms inter-trial interval (ITI) was inserted before the beginning of the next trial. The experiment included 3 blocks. Within each block, the 3 digits within each size condition were presented four times in each of the prime type conditions, yielding a total of 24 trials per block (12 ‘near’ and 12 ‘far’ trials), and an overall total of 72 trials in the experiment. The order of trials within each block was determined randomly. Prior to the beginning of the experiment participants completed a practice block, to ensure that they were proficient with the response mapping. As mentioned above, we hypothesized that a word representing proximity (i.e., small distance) would mainly prime small target digits, whereas a word representing remoteness (i.e., large distance) would mainly prime big target digits.

### Results

**Fig 3:**
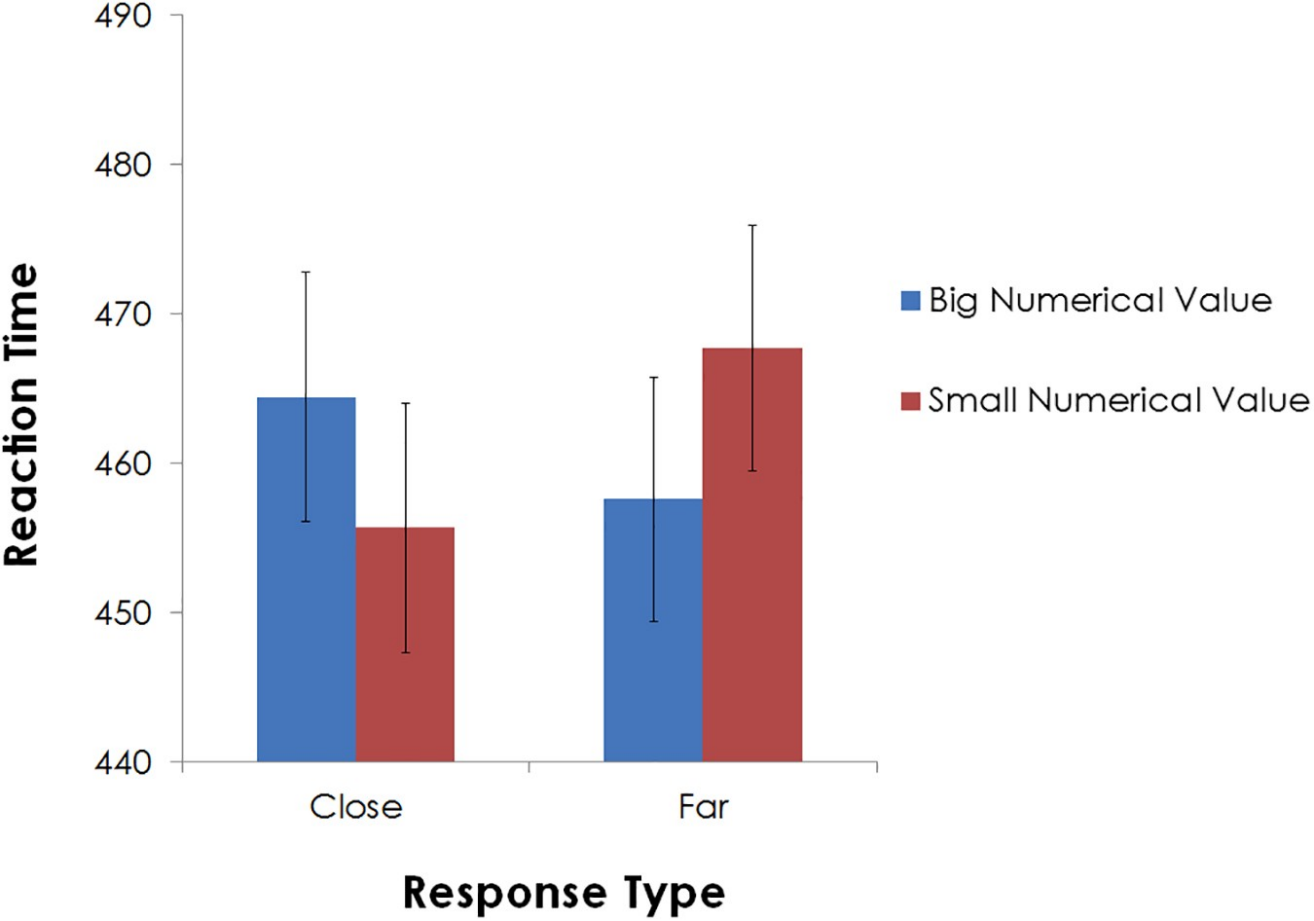
RTs in Experiment 2 as a function of prime type and numerical value. Standard errors are computed for the difference RT score (between big and small numerical values) [19], within each of the prime type conditions.

Accuracy rate was very high, and yielded an average of 99%. Trials yielding extreme outlier responses (i.e., RTs that deviated from participants’ mean RT by more than three standard deviations) were excluded from the RT analysis (less than 1%). Fig 3 presents participants’ RTs as a function of prime type and numerical value. A two-way analysis of variance (ANOVA) with prime type (‘near’, ‘far’) and target numerical value (small, big) as factors revealed no significant main effects (all Fs < 1), however, a statistically significant interaction effect was obtained, F(1, 24) = 4.53, p < .05, η^2^_p_ = .16. As in Experiment 1, the interaction revealed a congruency effect between conceived distance and numerical value, that is, shorter RTs for trials in which the two factors depicted magnitudes in the same direction (e.g., far, big numerical value), than in different directions (e.g., far, small numerical value) (none of the simple main effects were statistically significant, p>0.1). These findings corroborate on our earlier results, suggesting that distance is associated with numerical value, even when the former is represented on a rather abstract, conceptual level, rather than a visual and/or an action-related one.

## General discussion

Numerical cognition has long been associated with a variety of magnitude representations, such as size, time and space. Interactive relations between size or time, and numerical value, have been largely documented [1,2,23], suggesting a shared a representational mechanism for the different magnitude systems [24]. In addition, ample evidence has revealed associative relations between space and number perception. In particular, numbers were found to be represented on a left-to-right mental scale, often termed as the ‘mental number line’ [3,4,25]. Several studies have also documented a vertically-oriented number representation, according to which small numbers are associated with low spatial locations, while large numbers are associated with high locations [6–9].

In the present study we investigated a potential link between numerical perception and a different type of spatial dimension, namely, the perceived distance between an observer and an object. Two experiments were conducted, in the first distance was manipulated via near-the-body and far-from-the-body manual responses, while in the second it was manipulated via conceptual labels associated with close and far distances. Both experiments revealed a strong linkage between distance perception and numerical value processing, where large distances were associated with big number magnitudes and vice versa. Collectively, our results suggest that distance magnitudes may be associatively linked to numerical magnitudes and may affect digit processing above and beyond the effects of visual size.

While the relations of size and numerical value have been long documented, effects of egocentric distance or depth on numeral perception have been scarcely investigated. One of the reasons for the lack of studies on this issue is the difficulty to disentangle size and depth (egocentric distance) factors within a visual display. As mentioned earlier, in a three-dimensional world the physical size of stimuli and their perceived depth are negatively correlated, while the perceived size and the conceptual size are positively correlated with perceived depth (given equal retinal size of close and far objects, e.g., in the Ponzo illusion). Studies investigating the effects of perceived size of digits or of conceptual size of real world objects on various magnitude variables (e.g., numerical value, physical size) have typically disregarded the potential effects of distance on these variables. Thus, for instance, it is possible that both perceptual size and egocentric distance factors contributed to the SiCE observed when presenting numbers of equal retinal size on a Ponzo-like background [15]. Similarly, while viewing images of real-world objects presented at equal retinal size, implicit depth perception (i.e., perceiving an object as being viewed from a close or a far point of view) may have contributed to various congruency effects associated with numerical value or with physical size [16,26,27]. Furthermore, size and depth perception may share similar (or partially overlapping) underlying processing mechanisms and may both be tightly linked to a wider, common magnitude representational network. Studies investigating the role of the parietal cortex, for instance, have highlighted the importance of this region in functions such as reaching and grasping, and more generally in the perception of egocentric distance and object size computation required for action performance [28–30]. Thus, while acting on an object, one must compute both its egocentric distance and its precise size in order to correctly grasp and manipulate it. Moreover, recent theories have linked the processing of space and size, mediated by the parietal lobe, with other functions such as quantity and time perception. In particular, it was argued that all of these processing abilities (including numeral perception) are linked by a common metric for action, based on a shared underlying neural system [2,5,24].

Interestingly, parameters such as size, distance and numeral processing may not only be linked to each other via dorsal cortical mechanisms, but also via ventral ones. Studies investigating the cortical representation within the parahippocampal cortex (PHC) in the ventral visual pathway, have implicated its role in encoding large objects, by demonstrating a bias of this region towards stimuli of large retinal [31,32], perceived [33] and conceptual [34–37] size factors. Other studies, however, have argued that the PHC is predominantly involved in spatial processing and/or navigation [32,38–41], whereby distance and depth perception play a critical role [42]. Furthermore, we have recently shown that this cortical region may not only be sensitive to size and depth factors, but also to numerical value [34]. The present study adds to the current literature behavioral evidence in support of an associative link between perceived distance and numerical processing, and in doing so further suggests a common mechanism for the processing of different magnitudes which often interact with each other.
